# ‘Choose Psychiatry’ goes virtual: experiences and learning from the online 2020 National Psychiatry Summer School

**DOI:** 10.1192/bjb.2021.42

**Published:** 2022-06

**Authors:** Patricia Vinchenzo, Nikki Nabavi, Derek K. Tracy

**Affiliations:** 1 Queen's University Belfast, UK; 2 University of Manchester, UK; 3 Oxleas NHS Foundation Trust, London, UK; 4 Institute of Psychiatry, Psychology & Neuroscience, King's College London, UK; 5 Department of Psychiatry, University College London, UK

**Keywords:** PsychSoc, psychiatry society, recruitment, summer school, virtual conference

## Abstract

**Aims and method:**

COVID-19 has forced many educational events to go ‘virtual’. We report on the first online student-run National Psychiatry Summer School (NPSS). Evaluation of the online format and content was undertaken through survey feedback from almost 400 attendees.

**Results:**

The NPSS positively affected attendees’ perceptions of psychiatry as a career choice. The virtual format was positively received, with benefits including breaking down traditional barriers of geography and cost.

**Clinical implications:**

Post-COVID-19, a hybrid future of mixed virtual and face-to-face events is likely. Our work shows the viability of this and unique gains it might offer, and offers experiential learning on challenges encountered for others who wish to trial further virtual conferences.

COVID-19 has presented many changes, including a rapid move to virtual teaching.^1^ Online conferences have become the ‘new normal’, providing numerous new challenges and opportunities for medical education.^2^ ‘Choose Psychiatry’ initiatives by the Royal College of Psychiatrists (RCPsych) have also adapted to these changes. The National Psychiatry Summer School (NPSS) was one such event, planned from the outset as a virtual event with RCPsych sponsorship and successfully hosted online over 2 days in July 2020. To our knowledge, it was the first virtual summer school led by medical students with collaboration across devolved nations of the UK.

The NPSS had three primary aims: (a) to promote psychiatry as a career; (b) to help compensate for clinical placements lost during the pandemic; (b) to reduce geographical, financial and time-frame barriers to attendance. The first of these has been a common theme for other initiatives.^3^ However, the other two reflect innovative attempts to offset losses accrued during the COVID-19 pandemic and tap into novel opportunities from this disruption. Two secondary aims were to explore underrepresented subspecialties within the undergraduate curriculum and to evidence high-quality medical student-led initiatives.

We report the practicalities and learning from hosting this online event, present feedback data on the content and novel format, and offer proposals for the future optimisation of student engagement and recruitment through technology. We also consider the future of advancement of hybrid models and events that will likely combine virtual and face-to-face aspects, much of which is applicable to other online conferences.

## Method

Free-text feedback and ratings on a range of questions were collected on both days of the NPSS and a total of 726 feedback forms were completed (383 for day 1 and 343 for day 2), accounting for just over 81% of attendees. Merging the survey data produced a total of 379 responses.

Participants gave informed consent for their anonymised data to be subsequently analysed and disseminated in future research and promotional materials. Ethical approval was not required as the purpose of the study was to evaluate and improve a service. No formal research methodology was used beyond descriptive analysis of the survey data.

### Survey data collection

The majority of attendees were based in the UK (347/379); of those specifying a country, 7 were from Wales, 15 from Northern Ireland, 28 from Scotland and 87 from England. International attendees came from (1 from each country unless otherwise stated): Albania, Brunei (2), Canada, Egypt, Hungary, India (2), Ireland (2), Kuwait, Malaysia, Oman, Pakistan (3), Poland, Romania, Saudi Arabia and the United Arab Emirates (3).

In total, 215 attendees were current medical students, 104 were sixth form students, 13 were doctors and 33 were current/post-graduate students in other fields, including biomedical sciences and psychology.

### Feedback responses

**Fig. 1:**
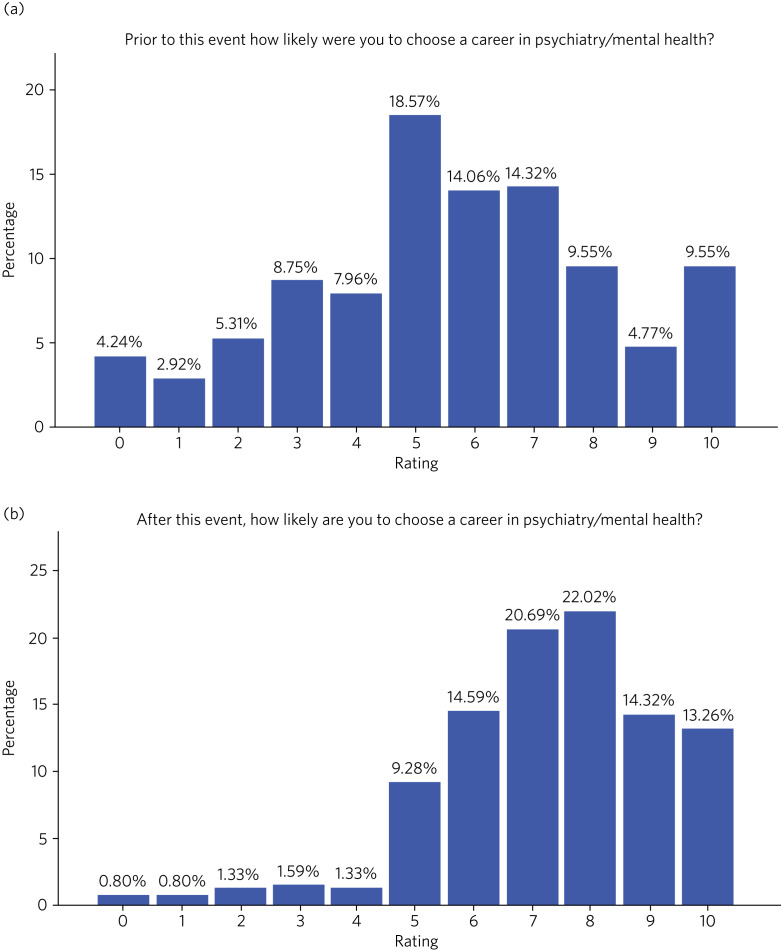
Attendee likelihood rating (a) before and (b) after event.

Attendees were asked to rate (on a scale of 0–10) how likely they were to choose a career in psychiatry/mental health before and after the event. The mean score increased from 5.66 (s.d. = 2.61) to 7.31 (s.d. = 1.95) (Fig. 1). They also completed a six-point questionnaire on various aspects of the event (Table 1). Feedback was very positive for the event; for example, across the 379 survey responses, over 99% of attendees ‘strongly agreed’ or ‘agreed’ that the presenters appeared enthusiastic about the subject and over 98% of attendees ‘strongly agreed’ or ‘agreed’ that, overall, this event was high quality.

**Table 1:** Subjective attendee (*n* = 379) ratings of the 2020 National Psychiatry Summer School

	Proportion of attendees selecting each response, %				
Statement	Strongly agree	Agree	Neutral	Disagree	Strongly disagree
‘The presenters appeared enthusiastic about the subject’	84.96	14.25	0.53	0	0.26
‘Overall, this event was of a high quality’	79.95	18.73	0.53	0.53	0.26
‘The event was well organised’	86.81	12.14	0.53	0.26	0.26
‘This event was successful in promoting psychiatry’	75.99	22.96	0.79	0	0.26
‘The event length was adequate’	56.73	33.51	7.92	1.58	0.26
‘There were enough breaks during the day’	38.79	40.63	11.87	7.92	0.79

### Free-text survey responses

Formal qualitative methods were not used, but free text was used to identify key themes in feedback from attendees. In total, 180/331 respondents from day 1 and 132/297 from day 2 provided qualitative free-text feedback. Attendees were asked whether there were ‘any other topics you wished we had covered?’. Popular responses included (but were not limited to) ‘Child and adolescent psychiatry’, ‘Perinatal psychiatry’, ‘Addiction psychiatry’, ‘LGBTQ mental health’, ‘Intellectual disability psychiatry’, ‘Psychotherapy’ and ‘Patients with lived experience’. Other responses referred to specific disorders or topics covered on day 2.

Attendees were asked to provide feedback to help improve future events, from which we identified 20 major themes: 10 on the format of the online event (Table 2) and 10 on its content (Table 3).

**Table 2:** Major themes regarding the online format of the 2020 National Psychiatry Summer School identified from qualitative data analysis

Format theme	Example quote
Accessibility	‘The fact that it was free and I was able to attend despite being from […] was very useful.’ (Theme 1)
Medical student hosts	‘I feel that the hosts played a massive part in making the day enjoyable and the way they went about changing it to suit the audience's needs, like the sixth former talk during lunchtime, would be something I am very thankful for. I am very inspired by the two of them.’ (Theme 2)
Quality	‘The event was incredibly well organised and done so much more professionally and with fewer technical problems than other virtual conferences arranged by larger organisations with more qualified staff.’ (Theme 3)
Organisation	‘I think the day was very well organised, it was truly one of the best events I have attended.’ (Theme 4)
Technology	‘It was amazing that you stuck to time and covered such a breadth of topics with speakers who were confident using the technology and extremely passionate about their subject areas.’ (Theme 5)
Social media use	‘It was great to get a good conversation going on Twitter – almost like networking (but not quite!).’ (Theme 6)
Interactivity	‘Although difficult to do, I think the talks could've been more interactive i.e. using the poll/voting tools available on Zoom.’ (Theme 7)
Providing post-event resources	‘Perhaps in future (with the permission of the speaker of course) it would be possible to record sessions so if some people are not able to make it they would be able to watch at a later date.’ (Theme 8)
Target audience	‘Perhaps there could have been different sessions available at different times/in breakout rooms at the end depending what stage you were in that you could choose to attend.’ (Theme 9)
Zoom fatigue	‘I would have wanted would be maybe a couple more breaks, just couldn't bear to miss any talks yet my eyes are definitely aching after a long day of screen-staring.’ (Theme 10)

**Table 3:** Major themes on the content of the 2020 National Psychiatry Summer School identified from qualitative data analysis

Content theme	Example quote
Speakers	‘Very well delivered webinars, speakers extremely interesting, enthusiastic, and knowledgeable about their subjects.’ (Theme 11)
Diversity	‘Diverse range of topics, and very glad to see that topics that aren't addressed enough (Islamophobia, Race, Equality) are being discussed and we can learn how, as medical students, to tackle these in healthcare and in our daily lives.’ (Theme 12)
Representation of subspecialties	‘I think the timetable has been very well developed to cover a wide variety of interesting topics that are otherwise neglected on many medical school's psychiatry curriculums’ (Theme 13)
Lived experience	‘Hearing about people's real lived experiences of mental health and working within the sector was really inspiring.’ 14
Patient simulation	‘I enjoyed the actor scenario and use of patient scenarios in the second and third lectures of the day. I always find this useful to put the information being given into the context of the real clinical world.’ (Theme 15)
Interest for non-psychiatrists	‘I loved it all, and that's coming from someone not actually pursuing psychiatry as an occupation.’ (Theme 16)
Compensation for lost experiences	‘Attending this summer school has re-lit the excitement in me about applying to medical school which seemed to have died down a bit in lockdown.’ (Theme 17)
Earlier exposure	‘At the University of […], we have psychiatry placements in our final year which were initially supposed to be early next year for me! It was great to get exposure to the speciality through the conference.’ (Theme 18)
Choose Psychiatry	‘I had slight doubts about choosing psychiatry but no more, this event has only deepened my passion for this career.’ (Theme 19)
College engagement	‘I hope you offer a summer school like this in future, and all the Royal [medical] colleges should do the same. I will look at signing up to the RCPsych now!’ (Theme 20)

## Discussion

### Practicalities of running the virtual 2020 NPSS

Psychiatry summer schools are one of the UK's Choose Psychiatry initiatives and one of many enrichment activities encouraging students to choose psychiatry.^4^ The first UK psychiatry summer school took place in 2009, organised by King's College London.^5^ These free-of-charge educational experiences have since expanded nationally (hosts have included Liverpool, Wessex and Leeds), ranging from one day to full week programmes.

**Fig. 2:**
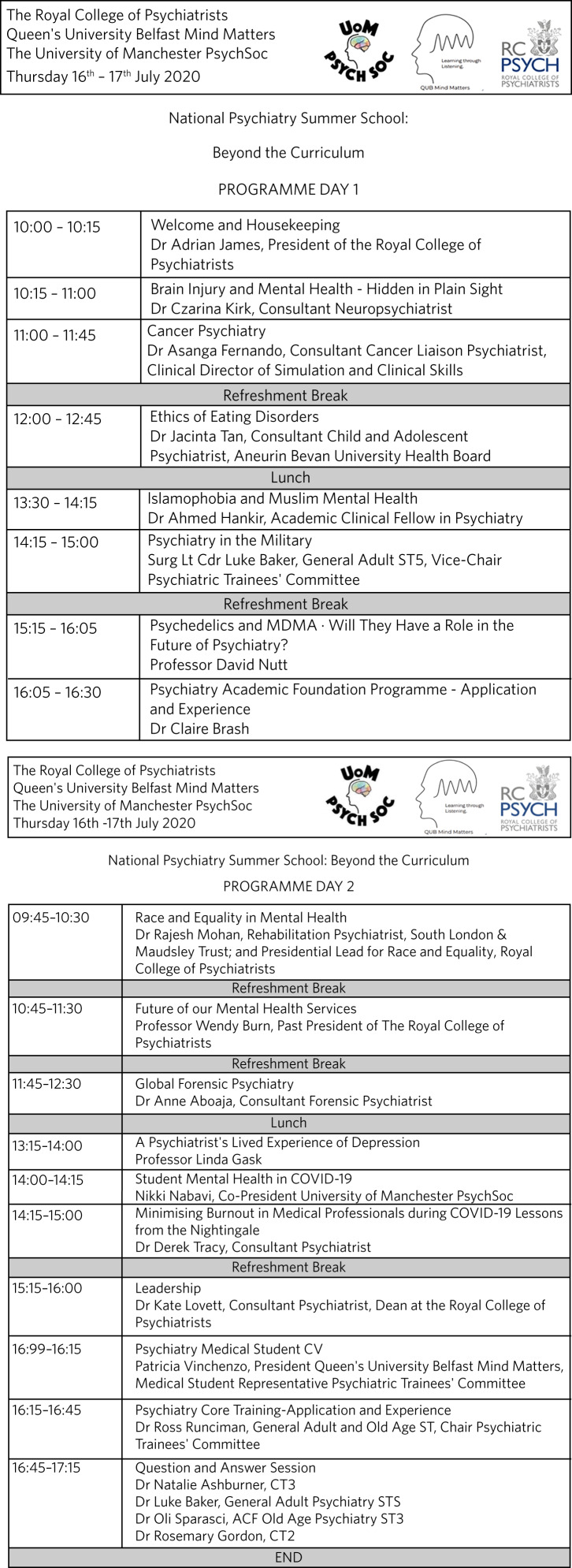
National Psychiatry Summer School 2020 programme.

The 2020 NPSS was not designed to make a profit, and funding for the online platform was provided by the RCPsych. The event was instigated, co-organised and co-hosted by the two medical student authors (P.V and N.N), who were PsychSoc Presidents at their respective universities, Queen's University Belfast and the University of Manchester, during this period. The programme was organised within approximately 1 month. It featured 18 diverse, eminent and award-winning doctors from a range of UK geographical locations, including RCPsych faculty staff. Thought was given to selecting speakers from subspecialties typically underrepresented on the undergraduate medical curriculum, such as eating disorders and global forensic psychiatry (Fig. 2). Speakers were ultimately recruited based on the organisers’ personal professional networks and experience of seeing speakers at previous face-to-face events. No speaker charges were incurred. The decision to use Zoom Video Webinar was made based on the hosts’ previous experience using the platform, which has control functions and security that allow attendees and panellists different privileges (speakers can share slides, sound and video without risk of interruption from the audience and it protects against the new phenomenon of ‘Zoom bombing’). For the NPSS, a 500-participant webinar license (£134.40 for that month) was added onto a pre-existing PsychSoc Zoom Pro meetings account (£14.39 a month).

Before the programme's release the event was advertised on Facebook, Twitter and Instagram using Queen's University Belfast and Manchester PsychSoc social media accounts. The event was open to all interested, including sixth formers as well as medical students. In total, 480 tickets, all free of charge, were available per day, with no restrictions or limitations in numbers by university or school. In total, 1029 expressed initial ‘interest’ in attending on Facebook, and the Eventbrite event page had 9747 views. Tickets were limited by Zoom platform capacity, and on both occasions all tickets were ‘sold out’ within 12 h. Tickets were released in two batches: the first on 29 June and the second 13 July 2020. The full programme was released on 11 July.

The 2020 NPSS had a total of 434 attendees log in overall on day 1 and 412 attendees on day 2.

### The online format: digital advantages and challenges

The 2020 NPSS has demonstrated that moving conferences online creates new challenges to overcome but can provide exciting novel opportunities. Organising a successful online conference can typically be achieved in a shorter time frame than similar in-person events. Once a suitable platform is chosen, there is no need to book a venue, catering, accommodation and so forth, and focus is solely on assembling the best possible programme. In comparison, the annual National Student Psychiatry Conference receives a minimum funding of £1500. Organisers are no longer constrained by geographical barriers (travel reimbursement costs and far greater time commitments) and the speaker pool is therefore much wider, time-zone permitting.

Moving conferences online can increase medical student and sixth form attendance and early career engagement, both nationally and internationally. Attendance at the NPSS overall was higher than at previous in-person National Student Psychiatry Conferences. Virtual forums can hold a greater capacity than a physical space at a much lower cost. The NPSS ‘sold’ 480 tickets. In comparison, previous National Student Psychiatry Conferences have sold approximately 122 tickets (Brighton and Sussex, 2018), 130 (Cardiff, 2019) and 156 (Bristol, 2020); historically, medical students from the host institution form the largest proportion of attendees at these face-to-face events. Cost access barriers to events are also overcome; attendees no longer incur travel expenses and can instead attend from the comfort of their own homes (theme 1 in Table 2), and attendance was particularly high for students from all three devolved nations when compared with the aforementioned conferences. The removal of travel costs, alongside the cost of accommodation, tickets and so forth contributes to an overall cost reduction for each attendee, helping to reach prospective doctors and future psychiatrists, especially in hard-to-recruit areas, and students from less advantaged backgrounds.

Attendees valued having medical student hosts and organisers (Table 2, theme 2). Prior to COVID-19 restrictions, collaboration between PsychSocs from two different countries was rare; but collaboration pooled perspectives from two institutions and networks, for both advertising and speaker recruitment. Consequently, there was an overall increased awareness of PsychSocs as a whole, and we predict future increase in medical student engagement with their local PsychSocs.

The quality and standard of an in-person event does not appear to have been lost – as reported in feedback (Table 2, theme 3). This included appreciation of keeping to programme timings, day structure and excellent organisation (theme 4). The hosts prompted the speakers on their available time and the appropriate number of audience questions, which was greatly valued by the audience, with comments such as ‘The event was incredibly well organised and done so much more professionally and with fewer technical problems than other virtual conferences arranged by larger organisations with more qualified staff’ (theme 3).

There are, however, challenges with online learning. Practice runs increased speaker confidence and minimised technical difficulties on the day, but the schedule was also purposefully designed to allow for some speaker delays and yet remain on time (Table 2, theme 5). Even with such precautions we experienced some technical difficulties on the day, including joining an online platform with inadequate internet connection or from a hospital/clinical trust from which access was blocked.

The attendees also appreciated the ‘buzz’ the conference had on Twitter (Table 2, theme 6). The NPSS ensured that all the speakers’ Twitter handles were made available to the attendees by displaying them on the screen in breaks.

Audience capacity varies across online platforms and payment schemes. Online free events may attract higher rates of ticket reservation, as attendees face no financial loss in not attending. Some who booked tickets did not attend and, conversely, some wished to attend but were not able to as all tickets had ‘sold out’. Organisers may wish to oversell ticket capacity, but to what extent this should be done is debatable, as there may be a risk of reaching attendee capacity on your platform and leaving some disappointed, particularly if there are some more popular talks from high-profile speakers. Several organisations livestream events across platforms but this may not be suitable for all. Interaction between attendees was limited, as the ‘chat’ function was disabled (Table 2, theme 7) because neither organiser had enough time to monitor this while managing other tasks.

We propose that recordings and slides should be made available to attendees following an event, but permission from each speaker must be sought and sensitive information removed (Table 2, theme 8). It has been suggested that distributing recordings post-event may reduce live attendance, but the extent of this is unknown. The use of breakout rooms and opportunities they can provide are limitless: perhaps icebreaker games, debates or concurrent workshops, which can allow for interactivity between attendees (but require a larger organisation committee to manage). Breakout rooms can target specific audiences, for example ‘psychiatry at medical school’ or ‘psychiatry within the foundation programme’ (Table 2, theme 9). Overall, we recommend targeting medical student and sixth form students separately to help meet the differing expectations and knowledge of these two groups while maintaining relevance, as shown by Wyke and colleagues.^6^

The length of the day and sessions should be considered: day 2 feedback suggests that sufficient activity-free breaks are welcomed and should be scheduled within the event programme (we had one additional break on the day 2 programme) to prevent ‘Zoom fatigue’ (Table 2, theme 10). Overall, attendance reduced throughout the day, but joining remotely provides attendees with flexibility to ‘dip in and out’ of the event, only attending for speakers they wish to hear.

### The content: maintaining quality speakers and topics in a ‘saturated marketplace’

Diverse, passionate and knowledgeable speakers are essential to convey the ethos of Choose Psychiatry (Table 3, theme 11). Attendees noted and appreciated the importance of the presented topics, including lived experience (theme 14), topics less commonly addressed (such as Islamophobia, race and equality) (theme 12) and subspecialties neglected on many medical schools’ psychiatry curriculums (theme 13). Attendees highlighted appreciation for the two medical student presentations (by P.V and N.N.), which were described as ‘more relatable’ and ‘easier to digest’. Virtual conferences may offer junior colleagues significantly more opportunities to present, and raising the voices of doctors from a variety of clinical standings and locations is equally important. Interactivity throughout the course of the two days included several speakers opting to include audience polls, as well as taking questions from the audience both during and after their talks. In addition, there was patient simulation, where Dr Fernando had invited an actor to play the patient while a medical student took their history (theme 15). Varying the options for student interaction, utilising audience polling, question platforms, chat features and the previously discussed breakout rooms are reported to maximise student engagement online.^7^

Feedback further highlighted the importance of patients’ mental health problems for all healthcare workers, not just prospective psychiatrists (Table 3, theme 16). For any medical specialty, it is vital for medical students to consider the mental health of their patients: parity of esteem and valuing mental health equally with physical health were key takeaway messages from the NPSS.

The 2020 NPSS was for some their first experience of psychiatry and/or medical education as a whole, as noted by the unanticipated but welcome large sixth form presence (not seen at past National Student Psychiatry Conferences). Feedback demonstrated how virtual events compensated for lost work experience and provided support for applications to medical school (Table 3, theme 17). Further support included providing medical students (a proportion of whom had psychiatry rotations cancelled or reduced because of COVID-19) with an insight into the specialty. Furthermore, the NPSS provided earlier, accessible psychiatry education to medical students who otherwise experience psychiatry rotations later in undergraduate clinical years (theme 18). Virtual psychiatry events may therefore help to mitigate the concerns regarding reduced psychiatry teaching and in turn engender future interest and boost recruitment.^8^

Lastly, the NPSS helped some students to consider pursuing psychiatry as a career (Table 3, theme 19), a notion further strengthened by ‘before and after’ ratings (Fig. 2). Following the event, there was a clear demand for future similar events and maintaining accessibility. Advertising these opportunities (such as becoming an associate member of the RCPsych) during programme breaks might sustain engagement and long-term recruitment (theme 20).

## Conclusions

COVID-19 created a need for online educational learning.^2^ Our survey data showed that the 2020 National Psychiatry Summer School had a positive impact on attendees’ perceptions of psychiatry as a career choice, and demonstrated how virtual medical education events can successfully engage large audiences while simultaneously reducing the historical geographical and financial barriers to beyond-curriculum teaching. The survey showed that the virtual format was positively received by our attendees.

The full potential of virtual events has yet to be fully realised, with a continuous evolution of, and learning from, innovative formats. Future work may further inform us of the benefits of virtual medical education events, and more formal qualitative and quantitative methods may be employed.

We believe there may be scope to run events that specifically target, engage and encourage sixth form students from all backgrounds; ‘schools only’ events offer a strong and fruitful possibility for psychiatry.^6^ Although these virtual events offer a myriad of opportunities, such as increasing access for medical students in the UK's devolved nations, it is important to recognise that they simultaneously reduce the networking opportunities. Post-COVID, the progress we have made with eco-friendly, online alternatives should not be lost, without compromising important aspects of face-to-face meetings that act as a ‘social glue’ in terms of networking.^9^ Events are most likely to encompass hybrid models and discussion remains on how these can be best utilised within psychiatry, medical education, recruitment and engagement. We must also recognise the risk of only certain groups being able to attend the in-person parts of hybrid events, with other groups ‘excluded’ from the social element of the events when attending virtually.

## Data Availability

The data that support the findings of this study are available from the corresponding author, P.V., upon reasonable request.

## About the authors

**Patricia Vinchenzo** is a medical student at Queen's University Belfast School of Medicine, Dentistry and Biomedical Sciences, UK. **Nikki Nabavi** is a medical student at the University of Manchester, Faculty of Biology, Medicine and Health, UK. **Derek K. Tracy** is a consultant psychiatrist and Clinical Director at Oxleas NHS Foundation Trust, London, and a senior lecturer in the Department of Psychosis Studies, Institute of Psychiatry, Psychology & Neuroscience, King's College London, UK.

## References

[1] Mark I, Sonbol M, Abbasian C. Running a Journal Club in 2020: reflections and challenges. BJPsych Bull [Epub ahead of print] 13 November 2020. Available from: 10.1192/bjb.2020.121. PMC7711334 33183387

[2] Nabavi N, Vinchenzo P, Tracy DK. “You're on mute”: how to organise a virtual medical conference. BMJ Student 2020; 371: m4942. 10.1136/bmj.m4942 33361383

[3] Pandian H, Mohamedali Z, Chapman GE, Vinchenzo P, Ahmed S, Mulliez Z, Psych Socs: student-led psychiatry societies, an untapped resource for recruitment and reducing stigma. BJPsych Bull 2020; 44: 91–5. 31950893 10.1192/bjb.2019.88 PMC8058822

[4] Halder N, Mulliez Z. Encouraging recruitment into psychiatry: practical initiatives. BJPsych Bull 2021; 45: 15–22. 32513344 10.1192/bjb.2020.53 PMC8058864

[5] Greening J, Tarn M, Purkis J. How to run a psychiatry summer school. Psychiatrist 2013; 37: 65–71.

[6] Wyke C, de Bernier G-L, Lam CC, Holt C, Butler S, Rajamani APR, Perspectives of GCSE students attending a psychiatry summer school in south London. BJPsych Bull 2021; 45: 114–9. 33762046 10.1192/bjb.2020.76 PMC8112016

[7] Gordon M, Patricio M, Horne L, Muston A, Alston SR, Pammi M, Developments in medical education in response to the COVID-19 pandemic: a rapid BEME systematic review: BEME Guide No. 63. Med Teach 2020; 42: 1202–15. 32847456 10.1080/0142159X.2020.1807484

[8] Sharma RK, Sharma B, Ogle HL. The recruitment legacy of COVID-19. BJPsych Bull 2020; 44: 177. 10.1192/bjb.2020.63 PMC8058853 32718374

[9] Sailsbury H. Helen Salisbury: mourning meetings. BMJ 2020; 371: m4177. 33144322 10.1136/bmj.m4177

